# The development and validation of the ‘Good Life in the Community Scale’ (GLiCS): a validation study with women migrants living in high income countries

**DOI:** 10.1186/s12889-022-12866-x

**Published:** 2022-03-11

**Authors:** C. F. Van der Boor, P. Christiansen, P. Anand, R. White

**Affiliations:** 1 Institute of Population, Health University of Liverpool Brownlow Hill, Liverpool, L69 3BX UK; 2 Economics, The Open University, London, MK7 6AA UK; 3 School of Psychology, Queen’s University Belfast, 8-30 Malone Road, Belfast, BT9 5BN Northern Ireland

**Keywords:** Wellbeing, Migration, Refugees, Economic migrants, Asylum seekers, Capability approach

## Abstract

**Background:**

To date, few assessment instruments have been developed to quantitatively measure the mental health status of migrant populations specifically. This paper describes the development and preliminary assessment of the ‘Good Life in the Community Scale’ (GLiCS). GLiCS is a wellbeing measure for migrant women in high-income settings that was coproduced with experts by experience across two phases.

**Methods:**

The study used a mixed-methods approach and was composed of two phases. *Phase I:* 88 initial items generated using qualitative data collected in a previous study were reduced to 42 through consultation with expert advisory panels, based on whether each item was considered understandable and relevant *Phase II:* these 42 items were piloted with a sample of migrant women (*N* = 109). A preliminary exploratory factor analysis was conducted using Oblique rotation. Internal consistency was measured using McDonald’s ω. Convergent validity was tested by correlating the GLiCS with the Oxford Capabilities Questionnaire Mental Health (OxCAP-MH), WHO-5 wellbeing index and Objective Social Outcomes Index (SIX). Incremental validity was tested using hierarchical regression analysis to ascertain the effect on the WHO-5 wellbeing index of: age, migration status, SIX, OxCAP-MH and GLiCS. Known groups validity, the ability a measure has to discriminate between groups likely to differ on the variables of interest, was tested between the different migrant categories using a simple between subjects ANOVA.

**Results:**

Exploratory factor analysis confirmed a 17-item (three-factor: (i) access to resources, (ii) belonging and contributing, (iii) independence) scale with high internal consistency (McDonald’s ω = 0.91). Convergent and incremental validity were also evidenced.

**Conclusion:**

The GLiCS has demonstrable good internal consistency and construct validity, and it presents a promising wellbeing measure for better understanding the experience of migrant women.

**Supplementary Information:**

The online version contains supplementary material available at 10.1186/s12889-022-12866-x.

## Background

Increasing numbers of people are leaving their country of birth because of conflict, poverty, unemployment, or in search of higher quality of life. The *International Organisation for Migration* (IOM) estimated that as of June 2019, the number of international migrants was almost 272 million globally [1]. Although migration is not a recent phenomenon, research into its impact on wellbeing and quality of life remains relatively sparse [2, 3]. Whilst many migrants have experienced multiple forms of trauma and life-threatening situations prior to and during the process of migration [4], more recently research attention has also recognized that the living conditions and post-migration stressors experienced in the settlement environment can exert an important influence on their mental health and wellbeing [5, 6]. Post-resettlement conditions have been suggested to be at least of equal importance for the mental health and wellbeing of migrants as pre-migratory conditions [7–9], including both forcibly displaced (i.e., asylum seekers and refugees, [10–12]), and non-forcibly displaced migrants (i.e., economic/labor migrants, [13]).

In particular, the United Nations High Commissioner for Refugees (UNHCR) underlines that some migrant subgroups are more disenfranchised than others, i.e. those with intersecting identities that may infer additional disadvantage including women/girls, children, persons with disabilities, sexual minorities and elderly men [14, 15]. The intersecting challenges, such as challenges related to gender, immigrant status and forced migration, might add up or even mutually reinforce each other [16], which can present multiple challenges to their overall integration, wellbeing and ability to live a full life post-resettlement [16–19].

To date, few assessment instruments have been developed to quantitatively measure the mental health status of migrant populations specifically. The majority of tools that have been developed have focused on (i) specific subgroups of migrants, most commonly asylum seekers and refugees, and (ii) measuring pre-migration sources of trauma and distress (i.e. The Harvard Trauma Questionnaire, [20]), or post-migration stressors and risk factors for mental ill-health (i.e. the Post-Migration Living Difficulties Scale; [21]; the Refugee Post-Migration Stress Scale, [22]), rather than positive mental health and/or wellbeing outcomes. There have been a number of calls in the literature to move away from the focus on psychopathology, and instead move towards broader outcomes relevant to psychosocial functioning in migrant groups [2, 23, 24]. It is argued that the dominant focus on trauma and distress overlooks other aspects of migrants’ mental health and wellbeing, for example relationships, sense of meaning [25] and sense of belonging [26]. This aligns with the recognition that mental health and wellbeing are not simply the absence of disease but instead encompass a wider understanding of what brings vitality into a person’s lived experiences, and that high levels of wellbeing and an understanding of positive predictors of mental health are necessary as well [27, 28]. As such, assessing levels of positive mental health and/or levels of wellbeing and identifying factors associated with higher levels of mental health and wellbeing has been highlighted [28, 29].

A recently conducted study used a participatory research approach to develop a wellbeing scale for a sample of newly resettled refugees from Myanmar and Bhutan in the USA [30]. The scale was developed in the context of an agricultural program aimed at strengthening health and improving wellbeing. The initial scale was composed of three subscales namely, (i) somatic experience, (ii) occupational balance, and (iii) social inclusion/self-identification. The authors acknowledged that “future iterations of survey development could include a factor analysis to measure the fit of a latent variable of wellbeing to the selected survey items, or correlation with similar, existing measures” [[30], p.27]. It is likely that assessment instruments of this kind could be beneficial for guiding policy, monitoring wellbeing, and identifying areas that require further support and attention for specific migrant groups post-resettlement.

### The capability approach

Sen’s Capability Approach (CA, [31]) is widely regarded to be of substantive importance for the conceptualization of multidimensional wellbeing [32, 33]. The CA holds that the wellbeing of a person ought to be assessed in the space of capabilities; the abilities to achieve the ‘beings and doings’ that they have reason to value in life [31]. From a CA perspective, human wellbeing depends on what resources enable people to do and to be. The ability to convert resources (e.g., social networks or education) into what people consider to be a *good life* varies and can include both health and non-health related variables like empowerment, relationships, participation, housing, and legal status [34]. As such, the CA not only assesses a person’s current circumstances, it also includes a focus on outcomes, agency and the individual’s substantive opportunities to achieve wellbeing [35].

The relevance and utility of applying the CA in the context of migration has been highlighted in a recent theoretical commentary by White & van der Boor [24]. The authors proposed the CA as a helpful framework to elucidate a focus on what living well means to migrant groups, understand what resources are available to these groups, and how these resources might interact with the persons’ capabilities and freedoms to engage in valuable functionings [24]. Factors operating at different levels of an individual’s social environment including their microsystem (i.e. factors that directly affect the individual), mesosystem (i.e. factors that impact on the social experience of the individual), exosystem (i.e. factors that are experienced by those in the person’s social networks) and the macrosystem (i.e. factors that operate at an institutional level) were highlighted as important when formulating an understanding of migrants’ experiences [24]. The authors concluded that individual choices, resources and entitlements will be highly influenced by people’s migration status [24, 36].

Significant attempts have been made to create evaluative tools and measures that are based on the CA. For example, the ‘*Human Development Index*’ published by the United Nations Development Program [37] is grounded in the understanding of development as a process of expanding individuals’ choices and opportunities. More recently, the *Oxford Poverty and Human Development Initiative* developed a specific measure of poverty [38], and the Organisation for Economic Co-operation and Development’s (OECD) *Better Life Index* which was launched in 2011 and aimed to measure the national wellbeing of OECD member countries [39]. However, there has been concern about the lack of available data relating to people’s actual capabilities, rather than the outcome of these capabilities (i.e., their functioning) ([40], for a review see Robeyns, [41]). Anand et al. [40] developed a list of over sixty capability indicators which could be used to generate information about an individual’s capabilities. This capability list was reduced and refined by Lorgelly et al. [42] into an 18-item capability wellbeing index (OCAP-18) and was validated for use in public health evaluations in Glasgow, UK with members of the public. Subsequently, Simon et al. [43] adapted the OCAP-18 to create the OxCAP-MH; a 16-item capability informed wellbeing measure for mental health research. The OxCAP-MH allows for the identification of capability domains most affected by mental illness and was validated on a sample of adults who had been involuntarily treated in hospital. To date, however, the CA has not been used to operationalize a measure of wellbeing for migrant populations.

A key objective of this paper is to describe the development of the ‘Good Life in the Community Scale’ (GLiCS) which was developed using the CA [31] as a guiding framework and coproduced with members of migrant populations in the United Kingdom (UK). To develop the items on the GLiCS, qualitative data collected in a previous study that explored what constitutes a ‘good life’ for female refugees in the UK from the perspective of the CA was used [44]. Specifically, the wording used by the participants to describe each domain relevant to achieving a ‘good life’ was extracted from the transcripts and used to create an initial draft of 88 individual items. In line with previous research that has highlighted the importance of liaising with experts by experience in the development of assessment instruments [45, 46], the current paper describes a multi-phase approach to the development of the GLiCS involving women with a lived experience of migration and/or supporting migrants. In addition, this paper also provides a preliminary assessment of the psychometric properties of the GLiCs.

## Methods

The current study used a mixed-methods approach and was composed of two phases: (i) Phase I: the refinement of the items of the GLiCS through consultation with women with a lived experience of migration and/or supporting those who do, and (ii) Phase II: the validation of the GLiCS. Ethical approval was granted by the Health and Life Sciences Research Ethics Committee of Psychology, Health and Society (approval reference number: 7561) at the University of Liverpool.

### Phase I: refinement of the ‘Good Life in the Community Scale’

The initial pool of items (GLiCS v0.1) which was developed by the lead author from the data gathered in a previous focus group study [44] was refined and checked for content validity through consultation with a *migration expert advisory panel*. This six-person panel consisted of four women who had experience of going through the asylum process and gaining a refugee status in the UK, and two female experts working with migrant women in the UK. The choice to include both members of the target population and experts working with migrant women was in line with suggestions made by Vogt and colleagues [47] to include ‘consultation with experts and members of the population’ (p.232) when assessing content validity. Similarly, Rubio et al. [46] emphasized the need to use a panel of experts who can provide constructive feedback on the quality of the measure, and objective criteria with which to evaluate each item. Recruitment to the panel was targeted to individuals who had previously been involved in the qualitative focus group study either by aiding recruitment or as participants themselves [44], and who have expressed a willingness to continue to be involved in the research. Participation was on a voluntary basis.

The six members recruited for the *migration expert advisory panel* were invited to participate via e-mail. The email highlighted that following on from the previous study [44], a wellbeing measure had been drafted and they were invited to individually review the items and provide written feedback. Upon agreeing to participate, a participant information sheet and consent form were sent. Following the provision of written consent, each participant received a copy of the GLiCS (v0.1; consisting of 88 items) via e-mail (see Appendix A) and were asked to provide written feedback. The form contained three statements for each individual item; (i) this item is clearly understandable to refugee women (ii) this item is relevant to the wellbeing of refugee women (iii) if not, how can the item be amended to ensure that it is clear and/or relevant? Questions (i) and (ii) were answered on a 5-point Likert scale (1—Strongly agree, 2- Agree, 3- Not sure, 4- Disagree, 5-Strongly Disagree). Question (iii) required a written response from participants. Additionally, participants were asked whether there were any additional questions or areas of wellbeing which should be included. Two participants did not complete the full Likert scales due to them having limited time, but provided written feedback on the items they considered needed changing. Following this feedback, an online discussion was hosted for debrief purposes.

**Fig. 1:**
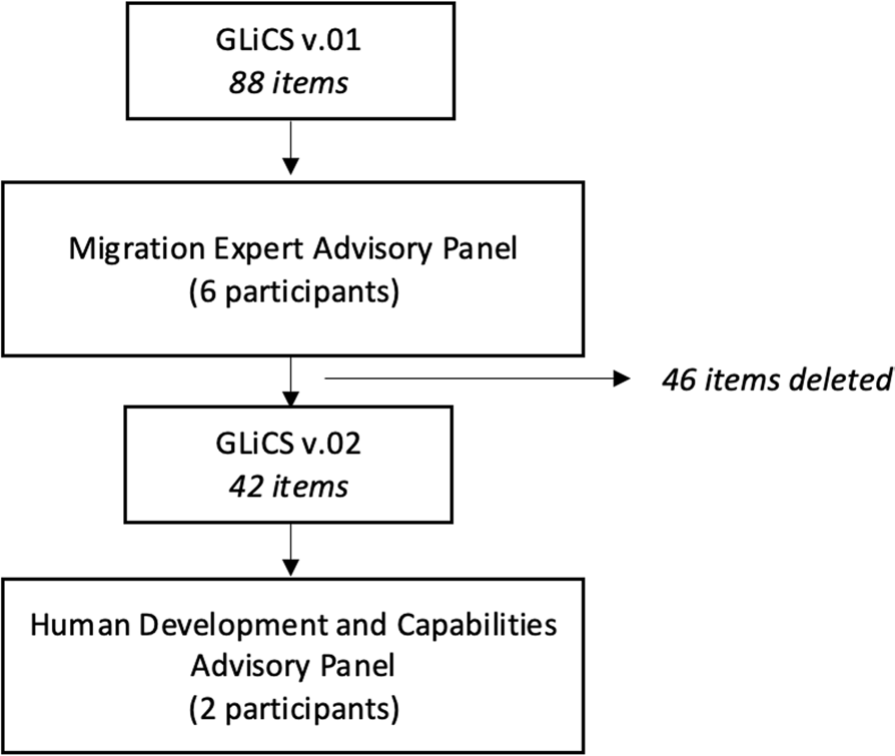
Diagrammatic representation of item deletion of the GLiCS

Based on the feedback provided all the items which obtained a relevance score of four or higher from any one reviewer was deleted. The only item exempt from this process of deletion was item ‘*I am able to do things to help me achieve a good level of mental health, for example talking about my worries or taking time to relax’* as this was a key theme that emerged from the previous research [44]. This led to a revised GLiCS (v0.2) of 42 items (see Fig. 1).

The GLiCS (v0.2) was then shared with two researchers belonging to the *Human Development and Capabilities Network*^1^ (HDCA) who have previous experience in developing capability-based measures. The HDCA is a global community of academic and practitioners that seeks to build an intellectual community around the ideas of human development and the CA and relate these ideas to the policy arena. These two researchers were contacted via e-mail and asked to review the list of items and suggest written amendments and/or refinements regarding the perceived clarity, relevance and the wording of each of the items in the context of measuring capabilities. The wording of specific items was adjusted according to the feedback collected. This resulted in GLiCS (v0.3).

Consistent with the approach taken by Lorgelly et al. [42], Simon et al. [43] and others in the development of their capability-informed assessment instruments, an ‘equal weights approach’ was used in the current study, whereby each item of the GLiCS received an equal rating. The equal weights approach has previously been adopted in the development of the Human Development Index [48], the Human Poverty Index and the Gender-related Development Index [49], the OECD Better Life Index [50], the OPHI Multidimensional Poverty Index [38], the OCAP-18 [42], and the OxCAP-MH [43].

### Phase II: factor analysis and validation

In Phase II, an exploratory factor analysis of the GLiCS (v.03) was carried out and a preliminary investigation of validity and internal consistency was conducted to determine whether the GLiCS is adequately able to measure capabilities in a sample of migrant women.

#### Design

This study used a cross-sectional design. Data was collected between August 2020 and March 2021. Participants were invited to complete the study on the online Qualtrics platform or verbally via a telephone consultation with author CB.

#### Participants

An initial validation of the GLiCS measure was conducted with a mixed group of females who identified as being a refugee, asylum seeker or economic migrant. The inclusion of three different migrant categories provided an opportunity to assess known groups validity; the ability a measure has to discriminate between groups likely to differ on the variables of interest [51].

A sample of adult woman (≥ 18 years) who identified as a refugee (definite leave to remain or settled status), asylum seeker or economic migrant living in the UK, New Zealand, or the Republic of Ireland who speak English were recruited to complete the survey. These countries were included due to the available networks of the researchers on the project. Three participants did not give full consent, two stated they were male, two participants confirmed they were not refugees, asylum seekers or economic migrants and were excluded, and one reported they did not live in a country relevant to this study, therefore these participants' data were deleted. Demographic questions were asked at the beginning of the survey (see Table 1). The final sample consisted of 109 women.

**Table 1:** Sociodemographic characteristics of sample (*N* = 109)

Characteristics	Sample (*N* = 109)
Self-reported Migration status	
Refugee	41(38%)
Asylum Seeker	19 (18%)
Economic Migrant	48 (44%)
Not stated	1 (0%)
Age	M = 34, IQR = 28—40
Country of Origin	
*Europe*	
Spain	17 (16%)
The Netherlands	13 (12%)
Poland	1 (1%)
*Asia*	
Iran	10 (9%)
Syria	9 (8%)
Pakistan	7 (6%)
Turkey	6 (6%)
Afghanistan	5 (5%)
Iraq	1 (1%)
Saudi Arabia	1 (1%)
Bangladesh	1 (1%)
Kurdistan	4 (4%)
*Africa*	
Nigeria	4 (4%)
Cameroon	2 (2%)
Sierra Leone	2 (2%)
Egypt	2 (2%)
Sudan	1 (1%)
Uganda	1 (1%)
East Africa	1 (1%)
*South America*	
Guatemala	1 (1%)
Colombia	1 (1%)
Venezuela	2 (2%)
Missing	17 (16%)
Participants with Children	60(55%)
Participants without Children	49 (45%)
Employment status	
Yes, regular employment	40 (37%)
Yes, in voluntary/protected/sheltered work	14 (13%)
No	55 (50%)
Housing	
Homeless or 24 h supervised	2 (2%)
Sheltered or supported accommodation	27 (25%)
Independent accommodation	80 (73%)
Living situation	
Living alone	35 (32%)
Living with partner or family	74 (68%)

#### Recruitment

Three different recruitment strategies were used; (i) advertisements inviting eligible people to take part in the research were disseminated online via dedicated social media sites (Twitter, Facebook, charity websites). The advertisement included a link and a QR code which could be scanned to access the measure online via the Qualtrics platform, (ii) service providers within organizations supporting migrants in the UK were asked to circulate information about the research project to potential participants, using the advertisement or a recruitment video in which author CB provided a verbal explanation of the study. Forty-five organizations/individuals agreed to disseminate the project via their networks. Lastly, (iii) every participant was invited to share the survey with other women in their networks who met the inclusion criteria. All participants were given the opportunity to enter a randomized draw to win one of four Amazon vouchers, three of which were worth £50 and one worth £100. Four randomized numbers were drawn from an online number generator.^2^

#### Measures

##### Objective Social Outcomes Index (SIX, [52])

The SIX is a brief index used for benchmarking social outcomes by capturing objective information about an individual’s social situation in three domains: employment, living situation and social contacts [52]. The instrument scores from 0 to 6 with higher scores indicating better outcomes. The SIX was used to test for convergent validity with the GLiCS. The internal consistency for the SIX on the current study was ω = 0.44. The low internal consistency in the current paper may be because the questions on the SIX are commonly collected as part of socio-demographic characteristics of participants, and do not test a construct per se [52].

##### Good Life in the Community Scale (GLiCS v0.3)

to the GLiCS (v0.3) is a 42-item measure which assesses whether migrant women judge their individual capabilities to be satisfied or deprived in the context of the UK. It contains forty-two items, and equal weights were assigned to each potential level of answers on a 5-point Likert scale; (1) strongly disagree, (2) somewhat disagree, (3) undecided, (4) somewhat agree, (5) strongly agree.

##### Oxford Capabilities questionnaire – mental health (OxCAP-MH, [43])

The OxCAP-MH is a wellbeing questionnaire developed within the conceptual framework of the CA. It was developed in the UK as a self-report measure for individuals with a severe mental illness [43]. It consists of 16 items rated on a 1–5 scale where higher scores indicate better capabilities. The OxCAP-MH demonstrated good internal consistency in the current study (McDonald ω = 0.86).

##### WHO-5 wellbeing index (WHO-5, [53])

The WHO-5 is a measure of wellbeing that owes its development from items of the Zung scales for depression, distress and anxiety [54] as well as from the General Health Questionnaire [55] and the Psychological General Well-Being Scale [56]. A key point of departure from these previous scales is that WHO-5 [53] only contains positively phrased items e.g. ‘I have felt cheerful and in good spirits’. The WHO-5 is comprised of 5 items rated on a 6-point Likert scale (i.e. 5- all the time, to 0-at no time). The WHO-5 demonstrated high internal consistency (McDonald ω = 0.95) and has been validated in a variety of settings.

#### Analysis

A mean imputation was carried out for three participants who had missing data for a maximum of two answers on the GLiCS. Participants with missing data on the WHO-5 and the OxCAP-MH were excluded from the analyses.

As the GLiCS data was ordinal (scored on a five-point Likert scale), a parallel analysis was conducted using the simulated polychoric correlation matrix to identify the number of likely components in the data. Following this, an exploratory factor analysis (EFA) was conducted on the polychoric matrix in order to determine the underlying factor structure. As factors were expected to be correlated, an Oblique rotation was applied [57]. Only items with a clear factor loading of 0.40 or higher [58, 59] were included in the GLiCS. Additionally, only cross loadings of less than 0.25 were used unless the item had a cross loading bigger than 0.60 [60, 61]. RStudio version 1.3.1093 was used to produce the GLiCS (v1.0), in particular using the Lavaan package.

The internal consistency of the GLiCS (v1.0) and each of the subscales was estimated by computing McDonald’s ω. To test the convergent validity, correlation analyses were performed to examine the associations between the GLiCS (v1.0) and theoretically related measures of wellbeing (WHO-5) and QoL (OxCAP-MH). Furthermore, the convergent validity was tested by investigating the correlations of the GLiCS (v1.0) with the SIX [52], as objective social outcomes such as employment and social contact would theoretically increase individuals’ capabilities. Finally, the incremental validity was tested using a hierarchical regression analysis to ascertain the effect on levels of wellbeing (WHO-5 wellbeing index; [53] of: age, migration status, objective social outcomes, OxCAP-MH and GLiCS (v1.0). The known groups validity was tested by running a simple one-way between subjects ANOVA to test the effect of migrant status on the GLiCS (v1.0) and the OxCAP-MH.

## Results

### Factor structure

Parallel analysis of the 42 item GLiCS (v0.3) suggested there were up to six underlying factors, this was used as an upper limit to the number of factors when exploring the structure in the EFA. The Kaiser–Meyer–Olkin measure suggested the sample was adequate (KMO = 0.50) and Bartlett’s test of sphericity demonstrated that correlations between the items were large enough for EFA (*χ*^2^ (861) = 6887.394, *p* < 0.001).

Factor one had an Eigenvalue of 5.82 (variance explained = 14%), factor two Eigenvalue = 4.91 (variance explained = 12%), factor three Eigenvalue = 5.29 (variance explained = 13%), factor four Eigenvalue = 5.10 (variance explained12 = %), factor five Eigenvalue = 3.32 (variance explained = 8%), factor six Eigenvalue = 1.57 (variance explained = 4%). The sixth factor identified by the parallel analysis had a substantially lower Eigenvalue and had one/no items loading onto it, therefore, a five-item solution was retained. Using a cut-off value of 0.40 [58, 59], we found six items loaded onto factor one, six items on factor two, five items on factor three, one item on factor four, and no items loaded onto factor five. A factor with fewer than three items is considered weak and unstable [62], therefore factors four and five were deleted and a three-factor solution was retained. The resulting 17-item GLiCS (v1.0) demonstrated good internal consistency (McDonald’s ω = 0.91). Each of the three factors constituted a meaningful subscale; (i) *access to resources,* (ii) *belonging and contributing,* and (iii) *independence;* each of which also demonstrated good internal consistency (see Table 2). See Appendix B for the full measure.

**Table 2:** Factor structure of the GLiCS (v 3.0)

	Rotated factors		
Scale item	Access to Resources	Belonging and Contributing	Independence
I am able to get sufficient money to meet my basic needs (through employment or benefits)	**0.87**	0.23	-0.04
I am able to buy essential items for myself when I want to, for example clothes, toiletries or things for my home	**0.90**	-0.02	0.06
I am able to access the kind of food that I would like to eat	**0.80**	-0.03	-0.07
I am able to access internet when I need to, for example on my phone or on a computer	**0.61**	-0.19	0.34
I am able to access courses to help build my skills and talents, for example art classes or dance classes	**0.55**	0.14	0.16
I am able to choose which city and neighborhood I want to live in	**0.58**	0.18	0.06
I am able to learn about my rights in this country, for example through support organisations	0.17	**0.48**	-0.06
I am able to feel I am a valued member of the community here	0.11	**0.46**	0.01
When people around me are feeling sad, I feel able to support them and make them feel more positive	0.05	**047**	0.9
I am able to rely on local organizations or charities for support with carrying out important tasks, for example paying bills or working through migration documents	0.10	**0.58**	-0.01
I am able to build a good life in this country	0.04	**0.79**	0.08
I feel happy about being in this country	-0.02	**0.78**	-0.24
I am able to read and write in the language of this country	0.27	-0.06	**0.65**
I am able to speak the official language(s) spoken in this country	0.16	-0.06	**0.62**
I am able to access green spaces in this country, for example parks or the countryside	-0.17	0.20	**0.51**
I am able to be involved in the decisions that affect my life, for example getting married or having children	0.13	-0.03	**0.59**
I am able to have my own privacy and keep information for myself if I want to, for example I can keep my bills and letters to myself	0.06	0.04	**0.63**
McDonald’s Omega	0.94	0.86	**0.82**

All significant loadings in bold

### Convergent validity

The convergent validity of the GLiCS (v1.0) was tested using the WHO-5 [53], the OxCAP-MH [43] and the SIX [52]. The overall scores for each measure can be found in Table 3. The GLiCS scores were correlated with wellbeing (WHO-5), capability-based wellbeing (OxCAP-MH), and with the SIX. Each of the subscales of the GLiCS were also correlated with the WHO-5, OxCAP-MH, and Six except subscale 2 which was not correlated with the SIX. For correlations see Table 4.

**Table 3:** The mean scores for each of the measures included in the analysis

Measure	Mean (SD)
SIX	3.67 (1.4)
OxCAP-MH	66 (13.20)
WHO-5	10.23 (6.55)
Total GLiCS	71.17 (12.33)
Subscale 1: Access to Resources	22.13 (6.94)
Subscale 2: Belonging and Contributing	22.86 (4.59)
Subscale 3: Independence	21.95 (3.30)

**Table 4:** Correlations between each of the scales and subscales used to test convergent validity

Variables	1	2	3	4	4	5	6
1. SIX	-						
2. OxCAP-MH	0.40*	-					
3. WHO-5	0.43*	0.54*	-				
4. Total GLiCS	0.56*	0.67*	0.61*	-			
5. Subscale 1: Access to Resources	0.64*	0.55*	0.51*	0.90*	-		
6. Subscale 2: Belonging and Contributing	0.18	0.46*	0.47*	0.72*	0.43*	-	
7. Subscale 3: Independence	0.42*	0.57*	0.47*	0.70*	0.53*	0.30*	-

^*^p < .001

### Incremental validity

To test the incremental validity of the GLiCS (v1.0), a hierarchical regression was run to analyze the effects of age, migration status (refugee, asylum seeker or economic migrant), SIX, OxCAP-MH and GLiCS on levels of wellbeing (WHO-5). Age, migration status and SIX were entered into step one of the model. At step 2, the OxCAP-MH was added, and the GLiCS was entered in step three. Variance inflation factors suggested multicollinearity was not a concern. The final regression model was significant and explained 42.7% of variance (F(5, 85) = 14.39, *p* < 0.001). Including the GLiCS (v1.0) at step three accounted for an additional 5.8% of variance in the model. Age, migration status and objective social outcomes (SIX) were not significant predictors of wellbeing (WHO-5). The OxCAP-MH (β = 0.25, *p* = 0.025) and GLiCS (β = 0.36, *p* = 0.003) were significant positive predictors of wellbeing. See Table 5.

**Table 5:** Hierarchical regression to test the effects of age, migration status, SIX, OxCAP-MH and GLiCS on levels of wellbeing (WHO-5)

Variable	Cumulative		Simultaneous	
	R^2^ Change	F-Change	β	*p*
*Step 1*	.24	9.18***		
Age			-.04	.667
Migration Status			.18	.050
*Step 2*	.16	22.36***		
SIX			.05	.652
OxCAP-MH			.25	.025
*Step 3*	.06	9.61**		
GLiCS			.36	.003

^***^p < .001, **p < .01

Lastly, a simple one-way between subject ANOVA was run to test the effect of migrant status (refugee, asylum seeker, economic migrant) on the GLiCS (v1.0) as a form of known groups validity [51]. The assumption of homogeneity of variances was not met (*p* < 0.001) therefore a Welch test was conducted. Welch’s test revealed a significant effect of migrant status on capability-based wellbeing (F(2, 47.77) = 26.92, *p* < 0.001). Tamhane’s post hoc tests revealed a significant difference between economic migrants (M = 77.08, SD = 7.99) and both refugees (M = 69.29, SD = 13.90, *p* = 0.007) and asylum seekers (M = 60.00, SD = 9.00, *p* < 0.001).

There was also a significant difference between asylum seekers and refugees (*p* = 0.009) with asylum seekers fairing worst on the GLiCS (v1.0) of all three groups followed by refugees and economic migrants respectively. These findings indicate that the GLiCS (v1.0) shows known groups validity for different migrant groups. The mean score on each of the three scales for each migrant group can be found in Table 6.

**Table 6:** Means, standard deviations, and *p* values for each subscale of the GLiCS depending on migration status

	Migrant Status			
Subscales	Asylum Seeker	Refugee	Economic Migrant	*p*
Subscale 1: Access to Resources	14.68 ± 6.57	21.29 ± 7.08	25.85 ± 3.75	< .001
Subscale 2: Belonging and Contributing	21.26 ± 5.08	22.80 ± 5.11	23.44 ± 3.80	.209
Subscale 3: Independence	19.74 ± 3.94	21.07 ± 3.46	23.50 ± 1.91	< .001

To determine whether this validity also exists for the OxCAP-MH, a simple one-way between subjects ANOVA was run to test the effect of migrant status (refugee, asylum seeker, economic migrant) on the OxCAP-MH. This analysis also revealed a significant effect of migrant status (F(2, 95) = 8.01, *p* = 0.001, η_p_^2^ = 0.14). Fisher’s Least Significant Difference post hoc test revealed a significant difference between refugees (M = 62.37, SD = 13.71) and economic migrants (M = 71.32, SD = 12.22, *p* = 0.002), with refugees scoring lower. There was also a significant difference between asylum seekers (M = 59.38, SD = 9.2) and economic migrants (*p* = 0.001), with asylum seekers scoring lower. No significant difference was found between the refugee and asylum-seeking groups (*p* = 0.421).

## Discussion

Over the last few years a number of calls have been made to expand the research focus in the area of migrant health to include psychosocial wellbeing and consideration of what factors may bring vitality to a person’s lived experiences [2, 23, 24]. The primary aim of this study was to coproduce a capability-based wellbeing measure for migrant women in high-income settings. An assessment instrument of this type will facilitate the measurement of capabilities of migrant women, which can have important implications for monitoring their mental health and wellbeing, better understanding predictors of positive outcomes, and identifying areas that require further support and attention.

The study was divided into two phases. In phase I, an 88-itemversion of the *Good Life in the Community Scale* (GLiCS v0.1) was reduced and refined to a 42-item version (v0.2) through consultation with a migration expert advisory panel made up of refugee women who had experienced the asylum system, women working with migrant populations, and two researchers with previous experience of developing a capabilities-based outcome measure. In phase II, a parallel analysis and EFA were carried out, which suggested a three-factor solution for the GLiCS (v1.0; henceforth to as ‘the GLiCS’). Each of these three factors constitutes a GLiCS subscale: *Access to resources* (6 items), *Belonging and contribution* (6 items), and *Independence* (5 items).

The preliminary validation of the GLiCS showed promising psychometric properties including high internal consistency and good convergent validity. The concurrent validity of the GLiCS was tested through a correlation analysis with the SIX. A moderate positive correlation was found, providing evidence for the concurrent validity. Incremental validity was assessed by determining whether the GLiCS significantly increased the amount of variance in wellbeing scores beyond that of the SIX and the OxCAP-MH (controlling for age and migration status). This was indeed the case. Furthermore, evidence of known groups validity was obtained for the GLiCS, as the measure revealed significant difference between the different migrant groups. Unlike the GLiCS, the OxCAP-MH did not discriminate between refugees and asylum seekers in terms of levels of wellbeing, suggesting the GLiCS is a more appropriate instrument for measuring capability-based wellbeing of migrant women in high-income settings. Overall, the GLiCS demonstrated good psychometric properties in the current sample.

Adding to the work of Logelly et al. [42], Greco et al. [63] and Simon et al. [43], the development of the GLiCS provides further evidence of the feasibility of operationalizing the CA in assessment of wellbeing. Importantly, the GLiCS is the first measure to be developed to measure capabilities in migrant populations. We believe that the three subscales that emerged from the data in the current study highlight the need to look across the different strata of the ecological model initially proposed by Bronfenbrenner [64]. Bronfenbrenner proposed an ecological theory of human development which placed individuals within multiple interacting systems including intra-individual, interpersonal, and larger social systems. These systems have previously been applied to understanding the mental health of migrant groups [i.e. [65–67]]. In the current study, the three scales highlight how a myriad of factors at different levels of the social environment of migrant women in the UK might affect their capabilities. When linking these subscales to Bronfenbrenner’s’ ecological model, the Access to Resources scale relates primarily to larger social systems, as the items within this subscale are influenced by the setting the individual finds themselves in (i.e., item 5; ‘*I am able to access courses to help build my skills and talents, for example art classes or dance classes*’). The *Belonging and Contributing* subscale speaks chiefly to the interpersonal system, i.e., pertaining to the social connections the individual can make within their community (i.e., item 2; ‘*I am able to feel I am a valued member of the community here’*). Lastly, the *Independence* subscale seems to relate to the intra-individual system i.e., the items speak to the person’s individual circumstances, sense of autonomy and agency (i.e., item 1. ‘*I am able to read and write in the language of this country*’). As such, the subscales of the GLiCS can help to shed light on what capabilities are being satisfied and/or deprived across the different levels of female migrants’ ecology post-migration. Moving forward, this could help inform interventions and forms of support aimed at increasing wellbeing in these populations. This was recently discussed in more detail in a commentary on enhancing the capabilities of forcibly displaced populations [24].

The development of the GLiCS can have important implications for policy and practice. Firstly, organizations (including non-governmental organizations and charities) supporting migrant women in high-income countries may benefit from using the GLiCS, as it can draw attention to specific issues that need to be addressed to support migrant wellbeing. It can also provide valuable information for advocacy efforts aimed at developing and amending policy and legislation relating to migration. Secondly, clinical services engaged in supporting the mental health and wellbeing of migrant women could benefit from using the GLiCS as an outcome measure to move beyond psychopathological outcomes and draw a more holistic picture of the individuals’ lived experience.

### Strengths, limitations and future directions

A major strength of this study is that it reports on the empirical development of the first CA-specific psychometric scale to be developed for and validated in a migrant population. At each stage of the assessment instrument’s development (including the previous qualitative work; [44] there was extensive involvement of experts by experience to ensure coproduction was facilitated. Following their participation, a number of participants provided positive feedback via e-mail to state that they had enjoyed participating and found the research highly relevant. A second strength of the study is the approach taken in the analyses. In previous studies researchers have erroneously used factor analyses developed for *interval-level* data, when the construct itself is *ordinal* in nature. To overcomes this specific statistical challenge, the current study used a polychoric correlation matrix [68].

However, there are some limitations to the current study. The limited sample size means there is an increased likelihood of errors of inference regarding the factor structure of this scale [62]. Best practice methods for EFA suggest a 10:1 subject to item ratio for EFA. This would suggest that for our initial 42-item GLiCS, a sample size of 420 was required. Given the challenges related to recruiting migrant women during the COVID19 pandemic, this desired sample size was not reached. As such, the conclusions presented here may not be generalizable beyond the current sample. Nonetheless, the EFA is designed and intended to be exploratory therefore the three-factor GLiCS presented in the current study can be used as a basis to conduct further analyses including confirmatory factor analysis, test–retest validity, and other latent variable modelling techniques that may help verify the proposed factor structure. This should also include exploring the association between the GLiCS with mental health measures such as the Patient Health Questionnaire (PHQ-9, [69]) and/or the Generalized Anxiety Disorder measure (GAD-7, [70]).If future research supports the psychometric properties of the GLiCS, then the assessment instrument could be used for evaluating the impact of resettlement and/or wellbeing interventions for migrants in high-income settings. This could provide insights into the benefits of interventions that go beyond health and basic resources, and instead provide a more holistic evaluation of wellbeing.

Beyond the limited sample size, the sample was also limited in terms of its representativeness of different migrant categories. This was particularly a concern for the EM given that the majority of EMs included in the sample came from the Netherlands and Spain. A recent report published by the Migration Observatory [71] reported that workers from the EU-14 countries are more likely to be in high-skilled employment in the UK than those from new EU member states (EU-8 and EU-2), who are more likely to be in low-skilled occupations. For future research it would be valuable to include a question on type of job and income level particularly for EM, to ensure a representative sample is achieved for this group, and includes EM in jobs classified as lower skilled.

Furthermore, the reliance on online recruitment due to the COVID19 national restrictions potentially excluded participants that do not have access to the internet and/or a smartphone. It is possible that these participants may have more limited capabilities and face more significant barriers to achieving high levels of wellbeing than those represented in the current sample. Similarly, the focus on participants who speak English excluded people from the current study. A future direction for the current research could be to translate the measure into other languages (e.g. Arabic) for use with participants who do not have a strong command of the English language. This could provide important insights into groups who may have more limited capabilities post-resettlement due to language barriers. Overall, the GLiCS should be subject to replication studies using diverse and representative samples.

Future research can also focus on adapting this measure to different groups. For example, future research might develop assessment instruments (or indeed adapt the GLiCS) for assessing the wellbeing of male migrants, different age groups, time duration of the migration status or migrant women in low and middle-income settings. Additionally, longitudinal research designs may be used to see how capabilities change as individuals go through the asylum process and gain a refugee status within specific contexts. This would shed light on how capability priorities and freedoms may change over time. A further area for future research would be to explore the relationships between the capabilities identified in this thesis and specific functionings (i.e. feeling integrated within the community or having personal agency). Within the CA, the distinction between capabilities and functionings is between the effectively possible (capabilities) and the realized outcome (functionings). This would include understanding the freedoms and opportunities that migrants have to lead the kind of life that they have reason to value, and subsequently assess the functionings they end up with in their lives post-resettlement.

## Conclusion

Our initial investigation into the psychometric properties of the GLiCS provides support for the internal consistency, validity and utility of the assessment instrument for assessing postmigration capability-based wellbeing for migrant women in high-income country settings. This is the first CA informed wellbeing scale to be developed and validated for use with migrant populations specifically. The three subscales found in the GLiCS (‘*accessing resources*’, ‘*contributing and belonging*’, and ‘*independence*’) highlight the different capability domains that are most relevant for migrant women to achieve high levels of wellbeing. The findings of this study provide further evidence of the merit, feasibility, and validity of operationalizing the CA for particular populations, and for applying the approach to outcome measure. The findings also highlight the relevance of developing a measure that speaks directly to the needs of migrant women in high-income settings.

## Supplementary Information

**Additional file 1.**

## Data Availability

Due to the nature of this research, participants of this study did not agree for their data to be shared publicly, so supporting data is not available.

## Abbreviations

IOM: International organisation for migration
UNHCR: United nations high commissioner for refugees
WHO: World health organisation
CA: Capability approach
UNDP: United nations development program
OECD: Organisation for economic co-operation and development
GLiCS: Good life in the community scale
UK: United Kingdom
HDCA: Human development and capabilities network
SIX: Objective social outcomes index
OxCAP-MH: Oxford capabilities questionnaire – mental health
WHO-5: WHO-5 wellbeing index

## Footnotes

1 HDCA website: https://hd-ca.org

2 Online number generator: https://numbergenerator.org/randomnumbergenerator/1-100#!numbers=3&low=1&high=99&unique=true&csv=&oddeven=&oddqty=0&sorted=false&addfilters = 
