# Supplementary material for: The development and validation of the ‘Good Life in the Community Scale’ (GLiCS): a validation study with women migrants living in high income countries
Source: BMC Public Health. 2022 Mar 11;22:486. doi: 10.1186/s12889-022-12866-x (PMC8917750; doi:10.1186/s12889-022-12866-x)
Supplement: Supplementary file 1 — Additional file 1. [file 12889_2022_12866_MOESM1_ESM.docx]

**Additional File 1.**

**Version 0.1 of the GLiCS focus group exercise**

Please read the following list of items and provide an answer for each item. If you consider an item to be unclear or irrelevant to measure well-being in refugee women then please provide a suggestion as to how the item can be amended, or whether it should be deleted altogether.

| **Item** | **Respondent’s Feedback** | | |
| --- | --- | --- | --- |
| Secure Legal Status | Please circle how much you agree or disagree with the statement | |  |
|  | This item is clearly understandable to refugee women | This item is relevant to the well-being of refugee women | If not, how can the item be amended to ensure that it is clear and/or relevant? |
| 1. I am able to progress plans to clarify my legal status, for example getting settled status or applying for a passport | Understandable:   1. Strongly agree 2. Agree 3. Not sure 4. Disagree 5. Strongly Disagree | Relevant:   1. Strongly agree 2. Agree 3. Not sure 4. Disagree 5. Strongly Disagree | Amendment: |
| 1. I am able to enjoy opportunities until I apply for a passport here | Understandable:   1. Strongly agree 2. Agree 3. Not sure 4. Disagree 5. Strongly Disagree | Relevant:   1. Strongly agree 2. Agree 3. Not sure 4. Disagree 5. Strongly Disagree | Amendment: |
| 1. My immediate family members have the appropriate approvals to travel in and out of this country at the current time | Understandable:   1. Strongly disagree 2. Disagree 3. Undecided 4. Agree 5. Strongly agree | Relevant:   1. Strongly disagree 2. Disagree 3. Undecided 4. Agree 5. Strongly agree | Amendment: |
| 1. I have the appropriate approvals to travel in and out of this country at the current time. | Understandable:   1. Strongly disagree 2. Disagree 3. Undecided 4. Agree 5. Strongly agree | Relevant:   1. Strongly disagree 2. Disagree 3. Undecided 4. Agree 5. Strongly agree | Amendment: |
| 1. At the current time I am free to do the same things host citizens do | Understandable:   1. Strongly disagree 2. Disagree 3. Undecided 4. Agree 5. Strongly agree | Relevant:   1. Strongly disagree 2. Disagree 3. Undecided 4. Agree 5. Strongly agree | Amendment: |
| 1. There are family members that I still want to have the opportunity to be reunited with. | Understandable:   1. Strongly disagree 2. Disagree 3. Undecided 4. Agree 5. Strongly agree | Relevant:   1. Strongly disagree 2. Disagree 3. Undecided 4. Agree 5. Strongly agree | Amendment: |
| 1. I am able to feel physically safe in this country | Understandable:   1. Strongly disagree 2. Disagree 3. Undecided 4. Agree 5. Strongly agree | Relevant:   1. Strongly disagree 2. Disagree 3. Undecided 4. Agree 5. Strongly agree | Amendment: |
| 1. I am able to sleep peacefully through the night in this country | Understandable:   1. Strongly disagree 2. Disagree 3. Undecided 4. Agree 5. Strongly agree | Relevant:   1. Strongly disagree 2. Disagree 3. Undecided 4. Agree 5. Strongly agree | Amendment: |
| 1. I am able to adhere to my cultural beliefs and practices in this country | Understandable:   1. Strongly disagree 2. Disagree 3. Undecided 4. Agree 5. Strongly agree | Relevant:   1. Strongly disagree 2. Disagree 3. Undecided 4. Agree 5. Strongly agree | Amendment: |
| 1. I feel protected by political institutions (such as the government) in this country | Understandable:   1. Strongly disagree 2. Disagree 3. Undecided 4. Agree 5. Strongly agree | Relevant:   1. Strongly disagree 2. Disagree 3. Undecided 4. Agree 5. Strongly agree | Amendment: |
| 1. I feel that my basic human rights are being respected in this country | Understandable:   1. Strongly disagree 2. Disagree 3. Undecided 4. Agree 5. Strongly agree | Relevant:   1. Strongly disagree 2. Disagree 3. Undecided 4. Agree 5. Strongly agree | Amendment: |
| 1. I am able to learn about my rights in this country | Understandable:   1. Strongly disagree 2. Disagree 3. Undecided 4. Agree 5. Strongly agree | Relevant:   1. Strongly disagree 2. Disagree 3. Undecided 4. Agree 5. Strongly agree | Amendment: |
| 1. I am comfortable using public resources when I need help, for example calling the police or an ambulance | Understandable:   1. Strongly disagree 2. Disagree 3. Undecided 4. Agree 5. Strongly agree | Relevant:   1. Strongly disagree 2. Disagree 3. Undecided 4. Agree 5. Strongly agree | Amendment: |
| 1. I am able to express my opinions and take part in the decisions that affect my life | Understandable:   1. Strongly disagree 2. Disagree 3. Undecided 4. Agree 5. Strongly agree | Relevant:   1. Strongly disagree 2. Disagree 3. Undecided 4. Agree 5. Strongly agree | Amendment: |
| 1. I am able to post what I want on social media, for example on Facebook, Instagram or Twitter | Understandable:   1. Strongly disagree 2. Disagree 3. Undecided 4. Agree 5. Strongly agree | Relevant:   1. Strongly disagree 2. Disagree 3. Undecided 4. Agree 5. Strongly agree | Amendment: |
| 1. I feel free to choose whether I want to practice a religion and am able to decide what religion I want to practice | Understandable:   1. Strongly disagree 2. Disagree 3. Undecided 4. Agree 5. Strongly agree | Relevant:   1. Strongly disagree 2. Disagree 3. Undecided 4. Agree 5. Strongly agree | Amendment: |
| 1. I am able to freely express my ideas and opinions | Understandable:   1. Strongly disagree 2. Disagree 3. Undecided 4. Agree 5. Strongly agree | Relevant:   1. Strongly disagree 2. Disagree 3. Undecided 4. Agree 5. Strongly agree | Amendment: |
| Accessibility of Resources |  |  |  |
| 1. In my current situation, I am able to access paid employment if I want to | Understandable:   1. Strongly disagree 2. Disagree 3. Undecided 4. Agree 5. Strongly agree | Relevant:   1. Strongly disagree 2. Disagree 3. Undecided 4. Agree 5. Strongly agree | Amendment: |
| 1. I am able to access the same work opportunities in this country as other people do (male or female) | Understandable:   1. Strongly disagree 2. Disagree 3. Undecided 4. Agree 5. Strongly agree | Relevant:   1. Strongly disagree 2. Disagree 3. Undecided 4. Agree 5. Strongly agree | Amendment: |
| 1. I am able to access employment that reflects the qualifications I have | Understandable:   1. Strongly disagree 2. Disagree 3. Undecided 4. Agree 5. Strongly agree | Relevant:   1. Strongly disagree 2. Disagree 3. Undecided 4. Agree 5. Strongly agree | Amendment: |
| 1. I am able to access my own money when I need to | Understandable:   1. Strongly disagree 2. Disagree 3. Undecided 4. Agree 5. Strongly agree | Relevant:   1. Strongly disagree 2. Disagree 3. Undecided 4. Agree 5. Strongly agree | Amendment: |
| 1. I am able to buy essential items for myself when I want to, for example clothes or things for my home | Understandable:   1. Strongly disagree 2. Disagree 3. Undecided 4. Agree 5. Strongly agree | Relevant:   1. Strongly disagree 2. Disagree 3. Undecided 4. Agree 5. Strongly agree | Amendment: |
| 1. I am able to access the kind of food that I would like to eat | Understandable:   1. Strongly disagree 2. Disagree 3. Undecided 4. Agree 5. Strongly agree | Relevant:   1. Strongly disagree 2. Disagree 3. Undecided 4. Agree 5. Strongly agree | Amendment: |
| 1. I am able to buy healthy food and have a balanced diet | Understandable:   1. Strongly disagree 2. Disagree 3. Undecided 4. Agree 5. Strongly agree | Relevant:   1. Strongly disagree 2. Disagree 3. Undecided 4. Agree 5. Strongly agree | Amendment: |
| 1. I am able to do the grocery shopping that I need | Understandable:   1. Strongly disagree 2. Disagree 3. Undecided 4. Agree 5. Strongly agree | Relevant:   1. Strongly disagree 2. Disagree 3. Undecided 4. Agree 5. Strongly agree | Amendment: |
| 1. I am able to access affordable healthcare in this country | Understandable:   1. Strongly disagree 2. Disagree 3. Undecided 4. Agree 5. Strongly agree | Relevant:   1. Strongly disagree 2. Disagree 3. Undecided 4. Agree 5. Strongly agree | Amendment: |
| 1. I am able to access healthcare services that are safe and effective | Understandable:   1. Strongly disagree 2. Disagree 3. Undecided 4. Agree 5. Strongly agree | Relevant:   1. Strongly disagree 2. Disagree 3. Undecided 4. Agree 5. Strongly agree | Amendment: |
| 1. I am able to access affordable medication when I need it | Understandable:   1. Strongly disagree 2. Disagree 3. Undecided 4. Agree 5. Strongly agree | Relevant:   1. Strongly disagree 2. Disagree 3. Undecided 4. Agree 5. Strongly agree | Amendment: |
| 1. I am able to access educational opportunities in my current situation | Understandable:   1. Strongly disagree 2. Disagree 3. Undecided 4. Agree 5. Strongly agree | Relevant:   1. Strongly disagree 2. Disagree 3. Undecided 4. Agree 5. Strongly agree | Amendment: |
| 1. I am able to access free courses in this country, for example language classes, art classes or dance classes | Understandable:   1. Strongly disagree 2. Disagree 3. Undecided 4. Agree 5. Strongly agree | Relevant:   1. Strongly disagree 2. Disagree 3. Undecided 4. Agree 5. Strongly agree | Amendment: |
| 1. I am able to do educational courses that can help me to share my ideas with other people | Understandable:   1. Strongly disagree 2. Disagree 3. Undecided 4. Agree 5. Strongly agree | Relevant:   1. Strongly disagree 2. Disagree 3. Undecided 4. Agree 5. Strongly agree | Amendment: |
| 1. I am able to read and write in the language of my host country | Understandable:   1. Strongly disagree 2. Disagree 3. Undecided 4. Agree 5. Strongly agree | Relevant:   1. Strongly disagree 2. Disagree 3. Undecided 4. Agree 5. Strongly agree | Amendment: |
| 1. I am able to speak the official language(s) spoken in this country | Understandable:   1. Strongly disagree 2. Disagree 3. Undecided 4. Agree 5. Strongly agree | Relevant:   1. Strongly disagree 2. Disagree 3. Undecided 4. Agree 5. Strongly agree | Amendment: |
| 1. I am able to improve my language skills in the official language(s) of this country if I want to | Understandable:   1. Strongly disagree 2. Disagree 3. Undecided 4. Agree 5. Strongly agree | Relevant:   1. Strongly disagree 2. Disagree 3. Undecided 4. Agree 5. Strongly agree | Amendment: |
| 1. I am able to receive support from local authorities and government agencies to access appropriate accommodation | Understandable:   1. Strongly disagree 2. Disagree 3. Undecided 4. Agree 5. Strongly agree | Relevant:   1. Strongly disagree 2. Disagree 3. Undecided 4. Agree 5. Strongly agree | Amendment: |
| 1. I am able to make plans to own my own residence in the future | Understandable:   1. Strongly disagree 2. Disagree 3. Undecided 4. Agree 5. Strongly agree | Relevant:   1. Strongly disagree 2. Disagree 3. Undecided 4. Agree 5. Strongly agree | Amendment: |
| 1. I am able to have a say in which city and neighborhood I want to live in | Understandable:   1. Strongly disagree 2. Disagree 3. Undecided 4. Agree 5. Strongly agree | Relevant:   1. Strongly disagree 2. Disagree 3. Undecided 4. Agree 5. Strongly agree | Amendment: |
| 1. I am able to use appropriate transportation to travel to the places that I wish to go to in this country, for example buses, trains, trams, etc. | Understandable:   1. Strongly disagree 2. Disagree 3. Undecided 4. Agree 5. Strongly agree | Relevant:   1. Strongly disagree 2. Disagree 3. Undecided 4. Agree 5. Strongly agree | Amendment: |
| Social support and integration |  |  |  |
| 1. When people around me are feeling sad, I am able to make them feel more positive by offering support | Understandable:   1. Strongly disagree 2. Disagree 3. Undecided 4. Agree 5. Strongly agree | Relevant:   1. Strongly disagree 2. Disagree 3. Undecided 4. Agree 5. Strongly agree | Amendment: |
| 1. I feel that my host society is tolerant, open and accepting of different people’s views | Understandable:   1. Strongly disagree 2. Disagree 3. Undecided 4. Agree 5. Strongly agree | Relevant:   1. Strongly disagree 2. Disagree 3. Undecided 4. Agree 5. Strongly agree | Amendment: |
| 1. I am able to contribute to discussions an/or plans aimed at rebuilding society in my country of origin | Understandable:   1. Strongly disagree 2. Disagree 3. Undecided 4. Agree 5. Strongly agree | Relevant:   1. Strongly disagree 2. Disagree 3. Undecided 4. Agree 5. Strongly agree | Amendment: |
| 1. I feel able to make friends and interact in my host community | Understandable:   1. Strongly disagree 2. Disagree 3. Undecided 4. Agree 5. Strongly agree | Relevant:   1. Strongly disagree 2. Disagree 3. Undecided 4. Agree 5. Strongly agree | Amendment: |
| 1. I my current situation I feel that I am able to be a valued member of the community | Understandable:   1. Strongly disagree 2. Disagree 3. Undecided 4. Agree 5. Strongly agree | Relevant:   1. Strongly disagree 2. Disagree 3. Undecided 4. Agree 5. Strongly agree | Amendment: |
| 1. I am currently able to spend time with people from my own cultural background | Understandable:   1. Strongly disagree 2. Disagree 3. Undecided 4. Agree 5. Strongly agree | Relevant:   1. Strongly disagree 2. Disagree 3. Undecided 4. Agree 5. Strongly agree | Amendment: |
| 1. In my host country I am able to meet and interact with people from different cultures | Understandable:   1. Strongly disagree 2. Disagree 3. Undecided 4. Agree 5. Strongly agree | Relevant:   1. Strongly disagree 2. Disagree 3. Undecided 4. Agree 5. Strongly agree | Amendment: |
| 1. In this country I am able to educate others about my cultural beliefs and practices | Understandable:   1. Strongly disagree 2. Disagree 3. Undecided 4. Agree 5. Strongly agree | Relevant:   1. Strongly disagree 2. Disagree 3. Undecided 4. Agree 5. Strongly agree | Amendment: |
| 1. I am able to feel respected and accepted by people from my host community | Understandable:   1. Strongly disagree 2. Disagree 3. Undecided 4. Agree 5. Strongly agree | Relevant:   1. Strongly disagree 2. Disagree 3. Undecided 4. Agree 5. Strongly agree | Amendment: |
| 1. I have the same opportunities to employment as citizens of this country | Understandable:   1. Strongly disagree 2. Disagree 3. Undecided 4. Agree 5. Strongly agree | Relevant:   1. Strongly disagree 2. Disagree 3. Undecided 4. Agree 5. Strongly agree | Amendment: |
| 1. I am able to look after my family members who are in this country or those who remain in my country of origin | Understandable:   1. Strongly disagree 2. Disagree 3. Undecided 4. Agree 5. Strongly agree | Relevant:   1. Strongly disagree 2. Disagree 3. Undecided 4. Agree 5. Strongly agree | Amendment: |
| 1. In my current situation I am able to feel supported by my family members | Understandable:   1. Strongly disagree 2. Disagree 3. Undecided 4. Agree 5. Strongly agree | Relevant:   1. Strongly disagree 2. Disagree 3. Undecided 4. Agree 5. Strongly agree | Amendment: |
| 1. I am currently able to receive support from a partner or significant other | Understandable:   1. Strongly disagree 2. Disagree 3. Undecided 4. Agree 5. Strongly agree | Relevant:   1. Strongly disagree 2. Disagree 3. Undecided 4. Agree 5. Strongly agree | Amendment: |
| 1. I am able to receive support from friends in this country | Understandable:   1. Strongly disagree 2. Disagree 3. Undecided 4. Agree 5. Strongly agree | Relevant:   1. Strongly disagree 2. Disagree 3. Undecided 4. Agree 5. Strongly agree | Amendment: |
| 1. I feel able and free to choose my own friends | Understandable:   1. Strongly disagree 2. Disagree 3. Undecided 4. Agree 5. Strongly agree | Relevant:   1. Strongly disagree 2. Disagree 3. Undecided 4. Agree 5. Strongly agree | Amendment: |
| I am satisfied with the opportunities that exist for migrants to support their children to integrate into their new setting | Understandable:   1. Strongly disagree 2. Disagree 3. Undecided 4. Agree 5. Strongly agree | Relevant:   1. Strongly disagree 2. Disagree 3. Undecided 4. Agree 5. Strongly agree | Amendment: |
| 1. I am satisfied with the opportunities that exist for migrants to be able to provide for their children | Understandable:   1. Strongly disagree 2. Disagree 3. Undecided 4. Agree 5. Strongly agree | Relevant:   1. Strongly disagree 2. Disagree 3. Undecided 4. Agree 5. Strongly agree | Amendment: |
| 1. I am satisfied with the opportunities that exist to support migrants to access affordable childcare | Understandable:   1. Strongly disagree 2. Disagree 3. Undecided 4. Agree 5. Strongly agree | Relevant:   1. Strongly disagree 2. Disagree 3. Undecided 4. Agree 5. Strongly agree | Amendment: |
| 1. I am satisfied with the support networks that migrants have to look after their children, for example family, friends or neighbor | Understandable:   1. Strongly disagree 2. Disagree 3. Undecided 4. Agree 5. Strongly agree | Relevant:   1. Strongly disagree 2. Disagree 3. Undecided 4. Agree 5. Strongly agree | Amendment: |
| 1. I am satisfied with the opportunities that exist for migrants to see their children happy in this country | Understandable:   1. Strongly disagree 2. Disagree 3. Undecided 4. Agree 5. Strongly agree | Relevant:   1. Strongly disagree 2. Disagree 3. Undecided 4. Agree 5. Strongly agree | Amendment: |
| 1. I am satisfied with the opportunities that exist for migrants to give their children a good education in this country | Understandable:   1. Strongly disagree 2. Disagree 3. Undecided 4. Agree 5. Strongly agree | Relevant:   1. Strongly disagree 2. Disagree 3. Undecided 4. Agree 5. Strongly agree | Amendment: |
| 1. I am satisfied with the opportunities that exist for migrants to see that their children are able to think about a future in this country | Understandable:   1. Strongly disagree 2. Disagree 3. Undecided 4. Agree 5. Strongly agree | Relevant:   1. Strongly disagree 2. Disagree 3. Undecided 4. Agree 5. Strongly agree | Amendment: |
| 1. I am able to get support from local organizations or charities to build a life in this country, for example support with meeting new people, making plans, getting used to a new system | Understandable:   1. Strongly disagree 2. Disagree 3. Undecided 4. Agree 5. Strongly agree | Relevant:   1. Strongly disagree 2. Disagree 3. Undecided 4. Agree 5. Strongly agree | Amendment: |
| 1. I am able to rely on local organizations or charities for support with carrying out important tasks, for example paying bills, working through migration documents, etc | Understandable:   1. Strongly disagree 2. Disagree 3. Undecided 4. Agree 5. Strongly agree | Relevant:   1. Strongly disagree 2. Disagree 3. Undecided 4. Agree 5. Strongly agree | Amendment: |
| Personal well-being |  |  |  |
| 1. I am able to exercise control over my living circumstances e.g. being able to live by myself if I want to. | Understandable:   1. Strongly disagree 2. Disagree 3. Undecided 4. Agree 5. Strongly agree | Relevant:   1. Strongly disagree 2. Disagree 3. Undecided 4. Agree 5. Strongly agree | Amendment: |
| 1. My age does not impact on me doing the things that matter to me. | Understandable:   1. Strongly disagree 2. Disagree 3. Undecided 4. Agree 5. Strongly agree | Relevant:   1. Strongly disagree 2. Disagree 3. Undecided 4. Agree 5. Strongly agree | Amendment: |
| 1. I am able to make my own informed choices regarding big life transitions, for example getting married or having children | Understandable:   1. Strongly disagree 2. Disagree 3. Undecided 4. Agree 5. Strongly agree | Relevant:   1. Strongly disagree 2. Disagree 3. Undecided 4. Agree 5. Strongly agree | Amendment: |
| 1. In my current situation I have the freedom to make my own decisions | Understandable:   1. Strongly disagree 2. Disagree 3. Undecided 4. Agree 5. Strongly agree | Relevant:   1. Strongly disagree 2. Disagree 3. Undecided 4. Agree 5. Strongly agree | Amendment: |
| 1. I am able to have the same status and respect as other members (male or female) of my household | Understandable:   1. Strongly disagree 2. Disagree 3. Undecided 4. Agree 5. Strongly agree | Relevant:   1. Strongly disagree 2. Disagree 3. Undecided 4. Agree 5. Strongly agree | Amendment: |
| 1. I am able to voice my opinions in my household and these opinions are respected | Understandable:   1. Strongly disagree 2. Disagree 3. Undecided 4. Agree 5. Strongly agree | Relevant:   1. Strongly disagree 2. Disagree 3. Undecided 4. Agree 5. Strongly agree | Amendment: |
| 1. I am free to choose what clothes I want to wear | Understandable:   1. Strongly disagree 2. Disagree 3. Undecided 4. Agree 5. Strongly agree | Relevant:   1. Strongly disagree 2. Disagree 3. Undecided 4. Agree 5. Strongly agree | Amendment: |
| 1. I am able to make my own choices and these choices are respected by other people living this country | Understandable:   1. Strongly disagree 2. Disagree 3. Undecided 4. Agree 5. Strongly agree | Relevant:   1. Strongly disagree 2. Disagree 3. Undecided 4. Agree 5. Strongly agree | Amendment: |
| 1. I am currently able to achieve a good level of physical health | Understandable:   1. Strongly disagree 2. Disagree 3. Undecided 4. Agree 5. Strongly agree | Relevant:   1. Strongly disagree 2. Disagree 3. Undecided 4. Agree 5. Strongly agree | Amendment: |
| 1. I am able to do things to help me establish a good level of physical health, for example going to the gym, going for a walk, dancing | Understandable:   1. Strongly disagree 2. Disagree 3. Undecided 4. Agree 5. Strongly agree | Relevant:   1. Strongly disagree 2. Disagree 3. Undecided 4. Agree 5. Strongly agree | Amendment: |
| 1. I am currently able to achieve a good level of mental health | Understandable:   1. Strongly disagree 2. Disagree 3. Undecided 4. Agree 5. Strongly agree | Relevant:   1. Strongly disagree 2. Disagree 3. Undecided 4. Agree 5. Strongly agree | Amendment: |
| 1. I am able to do things to help me establish a good level of mental health, for example talking to someone when I feel sad, or doing activities which make me happy | Understandable:   1. Strongly disagree 2. Disagree 3. Undecided 4. Agree 5. Strongly agree | Relevant:   1. Strongly disagree 2. Disagree 3. Undecided 4. Agree 5. Strongly agree | Amendment: |
| 1. I am able to feel good and confident about myself | Understandable:   1. Strongly disagree 2. Disagree 3. Undecided 4. Agree 5. Strongly agree | Relevant:   1. Strongly disagree 2. Disagree 3. Undecided 4. Agree 5. Strongly agree | Amendment: |
| 1. In my current situation I am able to feel free from emotional worry, stress and anxiety | Understandable:   1. Strongly disagree 2. Disagree 3. Undecided 4. Agree 5. Strongly agree | Relevant:   1. Strongly disagree 2. Disagree 3. Undecided 4. Agree 5. Strongly agree | Amendment: |
| 1. I am able to have a balance in my day to day routine | Understandable:   1. Strongly disagree 2. Disagree 3. Undecided 4. Agree 5. Strongly agree | Relevant:   1. Strongly disagree 2. Disagree 3. Undecided 4. Agree 5. Strongly agree | Amendment: |
| 1. I am able to live a happy life | Understandable:   1. Strongly disagree 2. Disagree 3. Undecided 4. Agree 5. Strongly agree | Relevant:   1. Strongly disagree 2. Disagree 3. Undecided 4. Agree 5. Strongly agree | Amendment: |
| 1. I am able to access green spaces in this country e.g. parks or the countryside | Understandable:   1. Strongly disagree 2. Disagree 3. Undecided 4. Agree 5. Strongly agree | Relevant:   1. Strongly disagree 2. Disagree 3. Undecided 4. Agree 5. Strongly agree | Amendment: |
| 1. I am able to enjoy leisure activities, for example going for walks, going to the cinema, visiting tourist attractions or listening to music | Understandable:   1. Strongly disagree 2. Disagree 3. Undecided 4. Agree 5. Strongly agree | Relevant:   1. Strongly disagree 2. Disagree 3. Undecided 4. Agree 5. Strongly agree | Amendment: |
| 1. I am able to access a place of worship if I want to e.g. a church, mosque, synagogue, etc | Understandable:   1. Strongly disagree 2. Disagree 3. Undecided 4. Agree 5. Strongly agree | Relevant:   1. Strongly disagree 2. Disagree 3. Undecided 4. Agree 5. Strongly agree | Amendment: |
| Hope for the future |  |  |  |
| 1. I am able to plan for my future in my current environment | Understandable:   1. Strongly disagree 2. Disagree 3. Undecided 4. Agree 5. Strongly agree | Relevant:   1. Strongly disagree 2. Disagree 3. Undecided 4. Agree 5. Strongly agree | Amendment: |
| 1. I feel confident that I will be able to achieve goals that I set. | Understandable:   1. Strongly disagree 2. Disagree 3. Undecided 4. Agree 5. Strongly agree | Relevant:   1. Strongly disagree 2. Disagree 3. Undecided 4. Agree 5. Strongly agree | Amendment: |
| 1. I am now able to have hope for the future | Understandable:   1. Strongly disagree 2. Disagree 3. Undecided 4. Agree 5. Strongly agree | Relevant:   1. Strongly disagree 2. Disagree 3. Undecided 4. Agree 5. Strongly agree | Amendment: |
| Independence |  |  |  |
| 1. I have the opportunity to be independent and autonomous in this country | Understandable:   1. Strongly disagree 2. Disagree 3. Undecided 4. Agree 5. Strongly agree | Relevant:   1. Strongly disagree 2. Disagree 3. Undecided 4. Agree 5. Strongly agree | Amendment: |
| 1. I am able to have my own privacy and keep information for myself if I want to | Understandable:   1. Strongly disagree 2. Disagree 3. Undecided 4. Agree 5. Strongly agree | Relevant:   1. Strongly disagree 2. Disagree 3. Undecided 4. Agree 5. Strongly agree | Amendment: |
| 1. I am able to build a good life in this country | Understandable:   1. Strongly disagree 2. Disagree 3. Undecided 4. Agree 5. Strongly agree | Relevant:   1. Strongly disagree 2. Disagree 3. Undecided 4. Agree 5. Strongly agree | Amendment: |
| 1. I feel able to fulfill my potential in this country | Understandable:   1. Strongly disagree 2. Disagree 3. Undecided 4. Agree 5. Strongly agree | Relevant:   1. Strongly disagree 2. Disagree 3. Undecided 4. Agree 5. Strongly agree | Amendment: |

Are there any additional questions or areas of wellbeing which you think we might have missed and should be included?

**Appendix B. The Good Life in the Community Scale**

| **Access to Resources** |  |
| --- | --- |
| I am able to get sufficient money to meet my basic needs (through employment or benefits) | 1. Strongly disagree 2. Somewhat disagree 3. Undecided 4. Somewhat agree 5. Strongly agree |
| I am able to buy essential items for myself when I want to, for example clothes, toiletries or things for my home | 1. Strongly disagree 2. Somewhat disagree 3. Undecided 4. Somewhat agree 5. Strongly agree |
| I am able to access the kind of food that I would like to eat | 1. Strongly disagree 2. Somewhat disagree 3. Undecided 4. Somewhat agree 5. Strongly agree |
| I am able to access internet when I need to, for example on my phone or on a computer | 1. Strongly disagree 2. Somewhat disagree 3. Undecided 4. Somewhat agree 5. Strongly agree |
| I am able to access courses to help build my skills and talents, for example art classes or dance classes | 1. Strongly disagree 2. Somewhat disagree 3. Undecided 4. Somewhat agree 5. Strongly agree |
| I am able to choose which city and neighborhood I want to live in | 1. Strongly disagree 2. Somewhat disagree 3. Undecided 4. Somewhat agree 5. Strongly agree |
| **Belonging and Contributing** |  |
| I am able to learn about my rights in this country, for example through support organisations | 1. Strongly disagree 2. Somewhat disagree 3. Undecided 4. Somewhat agree 5. Strongly agree |
| I am able to feel I am a valued member of the community here | 1. Strongly disagree 2. Somewhat disagree 3. Undecided 4. Somewhat agree   5 Strongly agree |
| When people around me are feeling sad, I feel able to support them and make them feel more positive | 1. Strongly disagree 2. Somewhat disagree 3. Undecided 4. Somewhat agree 5. Strongly agree |
| I am able to rely on local organizations or charities for support with carrying out important tasks, for example paying bills or working through migration documents | 1. Strongly disagree 2. Somewhat disagree 3. Undecided 4. Somewhat agree 5. Strongly agree |
| I am able to build a good life in this country | 1. Strongly disagree 2. Somewhat disagree 3. Undecided 4. Somewhat agree   5 Strongly agree |
| I feel happy about being in this country | 1. Strongly disagree 2. Somewhat disagree 3. Undecided 4. Somewhat agree 5. Strongly agree |
| **Independence** |  |
| I am able to read and write in the language of this country | 1. Strongly disagree 2. Somewhat disagree 3. Undecided 4. Somewhat agree 5. Strongly agree |
| I am able to speak the official language(s) spoken in this country | 1. Strongly disagree 2. Somewhat disagree 3. Undecided 4. Somewhat agree 5. Strongly agree |
| I am able to access green spaces in this country, for example parks or the countryside | 1. Strongly disagree 2. Somewhat disagree 3. Undecided 4. Somewhat agree 5. Strongly agree |
| I am able to be involved in the decisions that affect my life, for example getting married or having children | 1. Strongly disagree 2. Somewhat disagree 3. Undecided 4. Somewhat agree 5. Strongly agree |
| I am able to have my own privacy and keep information for myself if I want to, for example I can keep my bills and letters to myself | 1. Strongly disagree 2. Somewhat disagree 3. Undecided 4. Somewhat agree 5. Strongly agree |
